# *ELF18-INDUCED LONG NONCODING RNA 19* attenuates PAMP-induced callose deposition by modulating *UDP-glycosyltransferase 71B6*-associated ABA levels

**DOI:** 10.1007/s00299-026-03720-0

**Published:** 2026-01-19

**Authors:** Jun Sung Seo, So-Young Jang, Moon-Joo Lee, Jimin Lee, Nuri Oh, Jin-Ho Kang, Jang-Kyun Seo, Moonhyuk Kwon, Hye Sun Cho, Choonkyun Jung

**Affiliations:** 1 Department of Crop Science and Biotechnology, Dankook University, Cheonan, 31116 Republic of Korea; 2 Department of International Agricultural Technology, Seoul National University, Pyeongchang, 25354 Republic of Korea; 3 Integrated Major in Global Smart Farm, College of Agriculture and Life Sciences, Seoul National University, Seoul, 08826 Republic of Korea; 4 Division of Applied Life Science (BK21 Four), Anti-Aging Bio Cell Factory Regional Leading Research Center (ABC-RLRC), Research Institute of Molecular Alchemy (RIMA), Gyeongsang National University, Jinju, 52828 Republic of Korea; 5 Crop Biotechnology Institute, Institutes of Green Bio Science and Technology, Seoul National University, Pyeongchang, 25354 Republic of Korea; 6 Plant Systems Engineering Research Center, Korea Research Institute of Bioscience and Biotechnology, Daejeon, 34141 Republic of Korea; 7 Department of Biosystems and Bioengineering, KRIBB School of Biotechnology, Korea University of Science and Technology, Daejeon, 34113 Republic of Korea

**Keywords:** PAMP, PTI, ABA, Glycosylation, Callose deposition, *ELENA19*, Natural antisense transcript, *UGT71B6*, *Arabidopsis thaliana*

## Abstract

**Key message:**

***Cis-natural antisense transcript ELENA19 attenuates PAMP-triggered immunity by modulating ABA- and PAMP-inducible UGT71B6 expression, resulting in increased ABA levels and reduced ET-dependent flg22-induced callose deposition in Arabidopsis.***

**Abstract:**

Long noncoding RNAs (lncRNAs) have emerged as crucial regulators of various biological processes. However, the roles of lncRNAs in pathogen-associated molecular pattern (PAMP)-triggered immunity (PTI) remain largely unexplored in plants. Based on our previous custom lncRNA array analysis of Arabidopsis seedlings treated with PAMPs (elf18 and flg22), we identified a novel *ELF18-INDUCED LONG NONCODING RNA*, *ELENA19*. In this study, we characterized the function of *ELENA19* as a natural antisense transcript of *UDP-glycosyltransferase 71B6* (*UGT71B6*), which is responsible for the glycosylation of abscisic acid (ABA). *ELENA19* expression was rapidly upregulated upon treatment with ABA or PAMPs (flg22 and elf18). Among the genes neighboring *ELENA19*, only *UGT71B6* was responsive to both ABA and PAMP treatments. *UGT71B6* expression was significantly attenuated in *ELENA19*-overexpressing (OX) plants compared to wild-type (WT) plants after PAMP or ABA treatment. *ELENA19* OX plants were hypersensitive to ABA during germination and had higher endogenous ABA levels than WT plants, suggesting that *ELENA19* down-regulates *UGT71B6* expression and enhances endogenous ABA levels. Flg22-triggered callose deposition was reduced, and the expression of ethylene (ET)-dependent Flg22-induced genes was significantly down-regulated in *ELENA19* OX plants compared to WT plants, confirming the antagonistic interaction between ABA and ET signaling in the flg22-mediated immune response. These results demonstrate that *ELENA19* attenuates PAMP-triggered immunity by modulating *UGT71B6* expression.

**Supplementary Information:**

The online version contains supplementary material available at 10.1007/s00299-026-03720-0.

## Introduction

In higher organisms, only 2–3% of RNA transcripts encode proteins (ENCODE project consortium 2012). In the past, non-coding regions of the genome were considered junk DNA because of the lack of evidence for transcription and protein-coding ability. Recent technological advances, such as tiling arrays and next-generation sequencing, have highlighted the importance of noncoding RNAs (ncRNAs) transcribed from noncoding regions (Bu et al. 2012). NcRNAs play significant roles in regulating gene expression and organizing the development and maintenance of complex organisms (Perkins et al. 2005; Ponting et al. 2009).

NcRNAs can be classified into housekeeping and regulatory ncRNAs. Housekeeping RNAs comprise ribosomal RNAs (rRNAs) and transfer RNAs (tRNAs). Regulatory ncRNAs are classified into small, medium, and long ncRNAs based on their length (Alvarez-Dominguez and Lodish 2017; Dahariya et al. 2019; Ponting et al. 2009). Short ncRNAs, such as microRNAs and small interfering RNAs (siRNAs), are small transcripts of 20–24 nucleotides. Medium ncRNAs, such as small nucleolar RNAs and small nuclear RNAs, are transcripts of 60–200 nucleotides. Long ncRNAs (lncRNAs) are transcripts of more than 200 nucleotides with a coding potential of less than 100 amino acids (Dahariya et al. 2019; Nagano and Fraser 2011). Advances in experimental and computational technologies have led to the deep mining of more transcribed sequences. According to NONCODE v5, 548,640 lncRNAs have been found in 17 organisms, but fewer than 354,855 lncRNAs have been annotated (Bu et al. 2012; Ma et al. 2013; Fang et al. 2018). LncRNAs can be classified into long intergenic ncRNAs, intronic ncRNAs, and natural antisense transcripts (NATs), depending on their location and orientation in the genome. Long intergenic ncRNAs are transcribed from intergenic regions. Intronic ncRNAs and NATs are transcribed from regions that overlap with protein-coding genes. Intronic ncRNAs are transcribed from intronic gene regions (i.e., between exons), and NATs are transcribed in the antisense direction of the sense-coding genes (Ma et al. 2013). NATs can be classified into head-to-head (5′ side overlap), tail-to-tail (3′ side overlap), embedded overlap (complete overlap), and trans overlap (imperfect complementarity) NATs (Rosikiewicz and Makalowska 2016; Wight and Werner 2013).

Recently, plant lncRNAs have been studied extensively. *COLDAIR*, *COLDWRAP*, and *COOLAIR* are well-known plant lncRNAs; they are expressed in a vernalization-dependent manner from the *FLC* locus, which encodes a repressor of flowering. *COLDAIR* is expressed from the first intron and *COLDWRAP* is expressed from the promoter region. These two lncRNAs are involved in PRC2 complex formation and the recruitment of H3K27 tri-methylation to repress the expression of *FLC* (Kim and Sung 2017; Kim et al. 2017). *COOLAIR* is a NAT expressed antisense to *FLC* and represses *FLC* expression by replacing H3K36 tri-methylation with H3K27 tri-methylation (Csorba et al. 2014; Zhu et al. 2015). *FLORE*, expressed antisense of *CDF5*, represses the expression of *CDF5* and other *CDF* genes (Henriques et al. 2017). In maize, the natural antisense transcript *cis-NATZmNAC48* has been shown to negatively regulate the expression of *ZmNAC48*, a NAC transcription factor involved in drought stress responses, potentially through a double-stranded RNA-dependent mechanism affecting stomatal closure (Mao et al. 2021). As demonstrated in the examples above, NATs are generally known to repress the expression of their cognate sense gene. However, the recently reported NAT of *MAF4*, *MAS*, recruits WDR5a (a core component of the COMPASS-like complex) and activates the expression of *MAF4* by increasing H3K4 tri-methylation, a positive epigenetic mark (Zhao et al. 2018). Additionally, *Ef-cd*, the NAT of *OsSOC1*, positively regulates *OsSOC1* expression, promoting nitrogen utilization and increasing the photosynthetic rate (Fang et al. 2019).

Plants have developed innate immune systems to cope with various pathogens, and pattern-triggered immunity (PTI) is the first layer of plant innate immunity. Pathogens have conserved pathogen-associated molecular patterns (PAMPs) that are recognized by membrane-bound pattern recognition receptors, ultimately leading to resistance to pathogen attack (Zipfel 2008). The most well-known PAMPs are flagellin (flagellin epitope: flg22), which is the main building block of eubacterial flagella, and elongation factor Tu (EF-Tu epitope: elf18). The former is recognized by FLAGELLIN-SENSING 2 and the latter by the EF-TU RECEPTOR (Bigeard et al. 2015; Gomez-Gomez and Boller 2000). The immune response involves various defense responses, including mitogen-activated protein kinase cascade activation, reactive oxygen species bursts, and plant hormone signaling (Zhou and Zhang 2020). *ELENA1* is a lncRNA involved in PTI signaling in response to PAMPs. *ELENA1* positively regulates PTI signaling by enriching the mediator subunit MED19a at pathogenesis-related gene 1 and removing the negative regulator FIBRILLARIN 2 (Seo et al. 2019, 2017).

Abscisic acid (ABA) is a stress hormone that responds to various environmental stresses, including biotic and abiotic stresses (Sah et al. 2016; Vishwakarma et al. 2017). The function of ABA in abiotic stress responses is well documented; the elevation of endogenous ABA levels and propagation of ABA signaling render plants resistant to abiotic stress by activating various tolerance mechanisms (Zhu 2016). In defense responses to pathogens, ABA plays an ambivalent role, acting as either a positive or a negative regulator of disease resistance by intervening at multiple levels of defense signaling (Asselbergh et al. 2008; Ton et al. 2009). The role of ABA in disease resistance remains obscure because of its ambivalent function, depending on the plant tissue and developmental stage, as well as the pathogen and disease stage (Mbengue et al. 2016; Stec et al. 2016; Ton et al. 2009).

Glycosyltransferases are found in all living organisms. They catalyze the biosynthesis of glycosidic bonds by transferring a glucose moiety from a donor to an acceptor. In plants, uridine diphosphate glycosyltransferases (UGTs) are involved in the inactivation of various hormones via glycosylation (Li et al. 2001). Thus, glycosylation plays an important role in maintaining plant homeostasis by regulating the activity, level, and location of crucial cellular metabolites. Phylogenetic analysis of the nine conserved motifs of Arabidopsis UGTs revealed that they can be divided into 12 main groups (Li et al. 2001). UGT71B6, UGT71B7, and UGT71B8 belong to the same UGT71B subgroup of enzymes that conjugate glucose to ABA to produce ABA-glucose ester (ABA-GE) in the cytosol. ABA-GE accumulates in the vacuole and apoplast and is considered a storage or transport form (Dong and Hwang 2014; Dong et al. 2014). ABA-GE can be hydrolyzed back to ABA by the two β-glucosidases, AtBG1 and AtBG2, which exist in the endoplasmic reticulum or vacuole, respectively (Lee et al. 2006; Xu et al. 2012). Transcript levels of *UGT71B7*, *UGT71B8*, and particularly, *UGT71B6*, were increased upon treatment with ABA, NaCl, or mannitol (Dong et al. 2014). RNA interference (RNAi) lines in which *UGT71B6*, *UGT71B7*, and *UGT71B8* were knocked down simultaneously were hypersensitive to exogenous ABA and high-salt stress, but resistant to osmotic stress. Upon overexpression of *UGT71B6*, the opposite phenotype was observed, and endogenous ABA levels were decreased, suggesting that *UGT71B6* plays a critical role in plant growth, development, and adaptive responses by reducing ABA levels in plant cells (Dong et al. 2014).

Through custom lncRNA microarray analysis of elf18-treated seedlings, we previously screened for upregulated lncRNAs, which we termed *ELENA*s (*ELF18-INDUCED LONG NONCODING RNA*s) (Liu et al. 2012; Seo et al. 2017). In the current study, we found that *ELENA19* and *UGT71B6* were responsive to both ABA and PAMP treatments. Therefore, we investigated how *ELENA19* regulates *UGT71B6* expression and the crosstalk between PTI and ABA.

## Materials and methods

### Plant materials and growth conditions

*Arabidopsis thaliana* ecotype Columbia-0 (Col-0) was used as a control. A transfer DNA (T-DNA) insertion *ugt71b6* knockout mutant (SALK_001713C) was obtained from the Arabidopsis Biological Resource Center. Plants homozygous for the T-DNA insertion were selected by genotyping progeny plants according to signal instructions (http://signal.salk.edu/). Reverse transcriptase polymerase chain reaction (RT-PCR) was utilized to confirm the absence of transcript expression in the homozygous plants. All plants were grown on 0.6% agar medium containing 1/2 Murashige and Skoog (MS) salts (Duchefa, Haarlem, Netherlands), 1% sucrose (Duksan, Ansan, South Korea), and 0.5 g/L MES hydrate (Sigma-Aldrich, St. Louis, MO, USA) in a growth chamber at 22 °C under a photoperiod of 16 h light/8 h dark provided by three-colored LED lights.

### Genotyping of Arabidopsis *ugt71b6* knockout mutants

Rosette leaves from the T-DNA insertion mutants were collected, and genomic DNA was extracted using Quick-Extract^™^ DNA Extraction solution (Lucigen, Middlesex, UK). The genomic DNA was used as a template for PCR using the Solg^™^ e-Taq DNA Polymerase kit (SolGent, Daejeon, South Korea). Genotyping was performed using the primers SALK_001713C_LP and SALK_001713C_RP for *ugt71b6* (nucleotide sequences are provided in Table S1). The T-DNA insertion in the chromosome was identified using the T-DNA border primer, LBb1.3 (5′-ATTTTGCCGATTTCGGAAC-3′). Independent homozygous T-DNA mutants were identified.

### Construction of binary vectors and generation of transgenic plants

To generate transgenic plants, complementary DNAs (cDNAs) of *ELENA19* were amplified from Col-0 by PCR using gene-specific In-Fusion Cloning primers (TaKaRa, Kusatsu, Japan). The entry clone of *ELENA19* was Gateway-cloned into pBA-DC, an overexpression vector carrying the cauliflower mosaic virus 35S promoter (Zhang et al. 2005), using LR Clonase^™^ II Enzyme mix (Invitrogen, Waltham, MA, USA). All constructs were confirmed by sequencing and transformed into *Agrobacterium tumefaciens* strain GV3101. Plants were transformed using the floral dip method (Zhang et al. 2006).

### PAMP and ABA treatments

To analyze the response of *ELENA19* and its putative target genes to PAMPs, 10-day-old seedlings grown on 1/2 MS solid medium were transferred to 1/2 MS liquid medium (pH 5.7) containing 1 µM elf18 or flg22 (both from EZBiolab, Carmel, IN, USA). To analyze the response to ABA, 10-day-old seedlings were transferred from 1/2 MS solid medium to 1/2 MS liquid medium (pH 5.7) containing 100 µM ABA (Duchefa, Haarlem, Netherlands). After vacuum infiltration for 5 min, the seedlings were incubated in 1/2 MS liquid medium (pH 5.7) containing 100 µM ABA under continuous white fluorescent light and harvested at various time points.

### Real-time RT-PCR (RT-qPCR) and conventional RT-PCR

Total RNA was extracted from Arabidopsis seedlings using a Hybrid-R RNA purification kit (GeneAll, Seoul, South Korea), followed by DNase I treatment. One microgram of total RNA was reverse-transcribed using M-MLV reverse transcriptase (Enzynomics, Daejeon, South Korea). As *ELENA19* and *UGT71B6* completely overlap over the full length, strand-specific RT-PCR is essential. For *ELENA19-* and *UGT71B6-*specific RT-PCR, strand-specific reverse primers (Tagged At3g21781_qPCR-R or Tagged UGT71B6_qPCR-R) with tag sequence were used for reverse transcription (Fedak et al. 2016), and other transcripts except *ELENA19* and *UGT71B6* were detected using oligo(dT)_20_. RT-qPCRs were performed using gene-specific primers or gene-specific/Tag primer combinations (see Supplementary Table 1 for primer sequences) with EvaGreen^™^ Real-time PCR Smart mix (Solgent, Daejeon, South Korea) on an AriaMx Real-time PCR instrument (Agilent, Santa Clara, CA, USA). Target transcript levels were normalized to *ACT2* expression levels. For conventional RT-PCR, e-Taq DNA polymerase (Solgent) was used. The thermal cycling conditions were as follows: 95 °C for 2 min, 40 cycles of 95 °C for 20 s (denaturation), 60 °C for 40 s (annealing), 72 °C for 1 min per 1 kb (extension).

### Seed germination assays

For the germination assays, the WT and transgenic plants were grown and the fully ripened seeds were harvested at the same time to minimize variability due to seed dormancy. Additionally, all seeds used in the germination assays were subjected to the same after-ripening period under identical storage conditions before the experiment. At least 50 sterilized seeds from each transgenic line and wild-type (WT) plants were used, and three biological replicates were performed. Seeds were sown on 1/2 MS solid medium supplemented or not with 150 mM NaCl (Fisher Scientific, Hampton, NH, USA) or 1 µM ABA and then incubated in a growth chamber at 22 °C under a photoperiod of 16 h light/8 h dark (Dong et al. 2014). The seed germination rate was evaluated every 12 h for 6 days. Germination was defined as radicle protrusions of at least 1 mm.

### Aniline blue staining

Aniline blue staining reveals callose structures that appear in plant tissues after PAMP treatment (Clay et al. 2009). The seedlings were grown on 1/2 MS solid medium and were transferred to plates containing 1/2 MS liquid medium supplemented with flg22 or elf18 on day 10. The plates were then sealed tightly to prevent contamination. Ten-day-old seedlings were decolored in ethanol/glacial acetic acid (3:1 v/v) under vacuum for 5 min and then placed on a shaking platform for 2 h until the leaves appeared slightly translucent. The seedlings were then incubated in sodium phosphate buffer (0.07 M, pH 9.0; 12.46 g/L Na_2_HPO_4_.2H_2_O; Sigma-Aldrich, St. Louis, MO, USA) for 30 min. After several water washes, the seedlings were incubated in sodium phosphate buffer (0.07 M, pH 9.0) containing 0.05% aniline blue (Sigma-Aldrich, St. Louis, MO, USA) for 1 h. Then, the aniline blue solution was discarded and the seedlings were washed twice with distilled water. Cotyledons were mounted on slides with 50% glycerol and observed using a Zeiss AX10 Imager A2 microscope (Carl Zeiss, Oberkochen, Germany) under UV illumination with a broadband DAPI filter set (excitation filter 365 nm, beam splitter 395 nm, emission filter 445 nm).

### Measurement of endogenous ABA levels

Three-week-old seedlings (~ 200 mg) were collected using sharp tweezers and fresh weight was measured. The tissues were frozen in liquid nitrogen and ground into a fine powder. Then, 500 μL of 80% (v/v) methanol was added and the mixtures were incubated at 4 °C in the dark overnight. The methanol extracts were centrifuged at 12,000 × *g* for 10 min to remove debris and the supernatants were transferred into pre-cooled fresh 1.5-mL Eppendorf tubes and vacuum-dried at 4 °C to evaporate the supernatant. The powder was dissolved in 500 μL of 1 × TBS buffer (Agdia, Elkhart, IN, USA). The ABA content was determined by competitive enzyme-linked immunosorbent assay (ELISA) with an anti-ABA antibody using the Phytodetek Immunoassay Kit for ABA (Agdia, Elkhart, IN, USA) per the manufacturer’s protocol. According to the manufacturer’s cross-reactivity specifications, the antibody shows 0% cross-reactivity with ABA-glucose ester (ABA-GE) and < 0.1% cross-reactivity with phaseic acid/dihydrophaseic acid.

### Statistical analysis

All data are represented as the mean value ± standard deviation. Each data value was separately compared to the control value to determine significantly differences using a t-test (*P < 0.05, **P < 0.01) or ANOVA followed by Tukey’s test. Data were analyzed using the Microsoft Excel or IBM SPSS software.

## Results

### *ELENA19* and *UGT71B6* expression are induced by flg22 and elf18

**Fig. 1:**
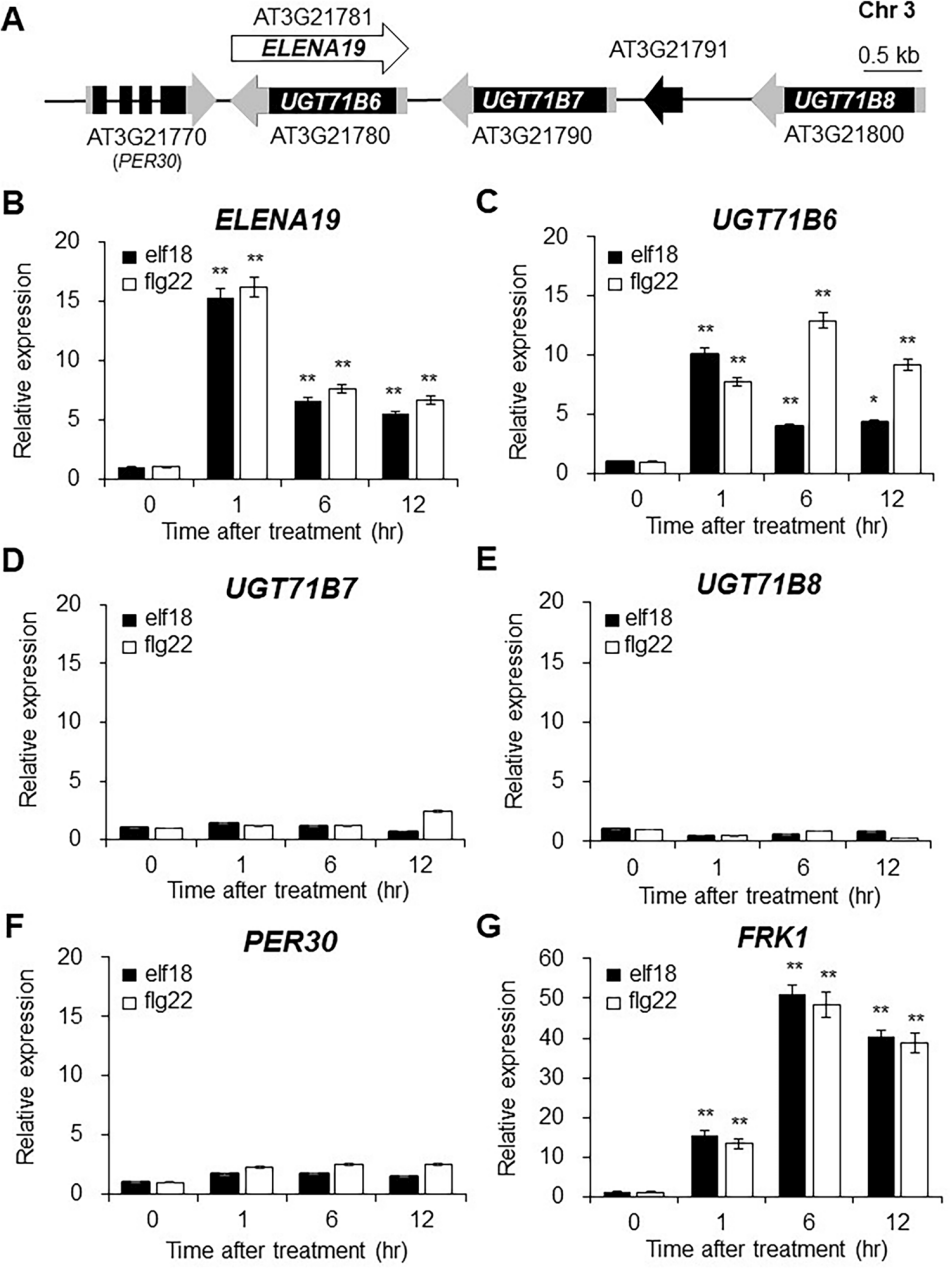
Expression pattern of *ELENA19* and its neighboring genes after flg22 and elf18 treatments. **A** Schematic representation of *ELENA19* (AT3G21781) and its neighboring genes. Gray and black boxes show untranslated regions and exons, respectively. Solid lines represent introns or intergenic regions. **B**–**G** Time-course expression analysis of *ELENA19* (**B**), *UGT71B6* (**C**), *UGT71B7* (**D**), *UGT71B8* (**E**), *PER30* (**F**), and *FRK1* (**G**) in 10-day-old Col-0 (WT) seedlings treated with 1 μM elf18 (black bar) or flg22 (white bar). Transcript levels were measured by RT-qPCR and normalized to *ACT2* expression levels. Data represent mean values ± SDs (n = 20 seedlings, at least three biological replicates). Significant differences from the no-treatment control (0 h) are indicated by asterisks (*P < 0.05, **P < 0.01, ANOVA followed by Tukey’s honestly significant difference test)

PAMP-responsive lncRNAs have previously been screened by custom microarray analysis of flg22- or elf18-treated Col-0 seedlings (Liu et al. 2012). Among 1,370 lncRNAs, 11 PAMP-responsive lncRNAs were validated using RT-qPCR analysis and were termed *ELENA*s (*ELF18*-*INDUCED LONG NONCODING RNA*s). *ELENA19* (AT3G21781) was annotated as a 1,616 nucleotide-long NAT of *UGT71B6* (AT3G21780) (Figure S1). To assess whether *ELENA19* is a bona fide lncRNA, we evaluated its coding potential using CPC2 (Kang et al. 2017). *ELENA19* showed a low coding probability score (0.239; below the 0.5 cutoff) and was therefore classified as noncoding. *UGT71B6* forms a gene cluster on chromosome 3 with *UGT71B7* (AT3G21790) and *UGT71B8* (AT3G21800). A pseudogene of *UGT71B7* (AT3G21791) is located between *UGT71B7* and *UGT71B8*. *PER30* (encoding a peroxidase superfamily protein; AT3G21770) is located near the 5′ end of *ELENA19* (Fig. 1A).

To analyze the response of *ELENA19* to PAMP treatment, 10-day-old WT (Col-0) seedlings were treated with 1 μM flg22 or elf18, and transcript levels were analyzed by RT-qPCR. *ELENA19* expression was rapidly induced, peaked within 1 h of treatment, and then gradually decreased (Figs. 1B and S2A). We next assessed the expression patterns of the neighboring genes, *UGT71B6*, *UGT71B7*, *UGT71B8*, and *PER30*, and found that they did not respond to elf18 and flg22 treatments, except *UGT71B6*; *UGT71B6* expression was also induced by elf18 and flg22 treatments (Fig. 1C–F; Figure S2B). *FRK1* was included as a representative PAMP response marker (Fig. 1G). *UGT71B6* has been reported to be a regulator of ABA homeostasis and is upregulated by ABA, NaCl, or mannitol treatment (Dong et al. 2014). Therefore, we examined *ELENA19* expression after ABA treatment. The results confirmed that it rapidly responds to ABA (Figure S2C). *UGT71B6* expression was also rapidly upregulated by ABA treatment (Figure S2D). These results demonstrated that *ELENA19* and *UGT71B6* rapidly respond to PAMPs and ABA, suggesting that they are involved in PTI and ABA signaling pathways.

### *ELENA19* down-regulates *UGT71B6* expression after flg22 treatment

**Fig. 2:**
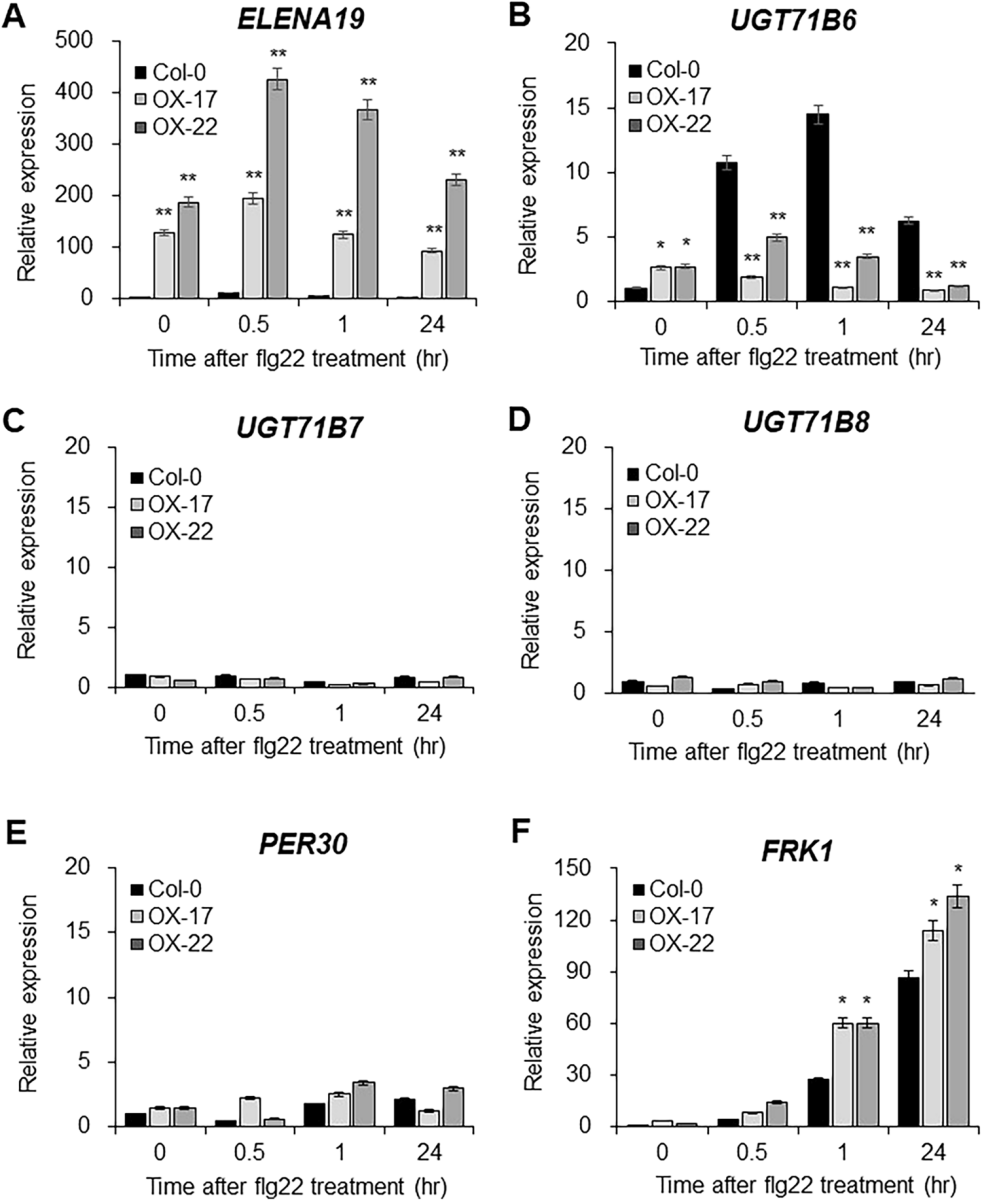
Expression analysis of *ELENA19* and its neighboring genes in WT and *ELENA19* OX plants after flg22 treatment. **A**–**F** Time-course expression analysis of *ELENA19* (**A**), *UGT71B6* (**B**), *UGT71B7* (**C**), *UGT71B8* (**D**), *PER30* (**E**), and *FRK1* (**F**) in 10-day-old seedlings treated with 1 μM flg22. Transcript levels were measured by RT-qPCR and normalized to *ACT2* expression levels. Data represent mean values ± SDs (n = 20 seedlings, at least three biological replicates). Significant differences from the no-treatment control (0 h) are indicated by asterisks (*P < 0.05, **P < 0.01, ANOVA followed by Tukey’s honestly significant difference test)

To understand the function of *ELENA19*, we generated *ELENA19*-overexpressing (OX) transgenic plants. The annotated full-length *ELENA19* transcript was amplified from cDNA and subcloned into a constitutive overexpression vector (Figure S3A). We determined *ELENA19* expression levels in the transgenic plants and selected two representative lines, OX-17 and OX-22 (Figure S3B). T3 transgenic plants were used in subsequent experiments. To determine whether *ELENA19* affects the expression of the neighboring genes via *cis*-regulation, we analyzed the expression of *ELENA19* and its neighboring genes in the WT and *ELENA19* OX lines after 1 μM flg22 treatment. The results confirmed that *ELENA19* was expressed at more than 100-fold higher levels in the OX lines than in Col-0 plants (Fig. 2A). *FRK1* was included as a marker gene. Interestingly, *UGT71B6* expression was significantly down-regulated in the OX lines compared to that in the WT after flg22 treatment, suggesting that *ELENA19* negatively regulates *UGT71B6* expression in response to flg22 (Fig. 2B). *UGT71B7* and *UGT71B8* expression did not differ between the WT and *ELENA19* OX lines, regardless of flg22 treatment. *PER30* expression showed variation and differed slightly, but not significantly, between the WT and OX lines (Fig. 2C–E). Although the nucleotide sequence similarity among *UGT71B6*, *UGT71B7*, and *UGT71B8* is high (~ 70% coding sequence identity), *ELENA19* was found to affect only *UGT71B6* expression, suggesting that it specifically regulates its target gene, *UGT71B6*.

### *ELENA19* down-regulates *UGT71B6* expression after ABA treatment

**Fig. 3:**
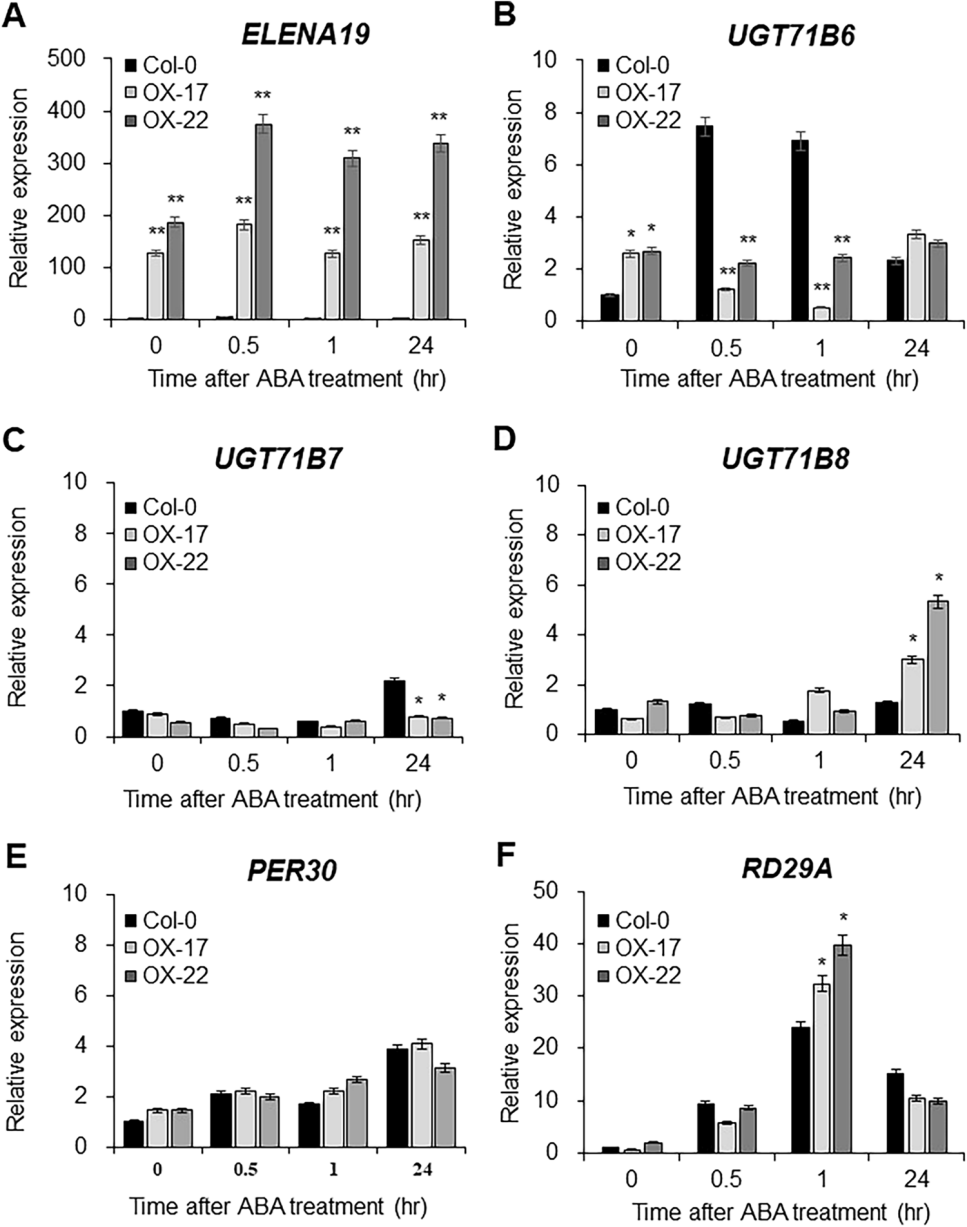
Expression analysis of *ELENA19* and its neighboring genes in WT and *35S:ELENA19* plants after ABA treatment. **A**–**F** Time-course expression analysis of *ELENA19* (**A**), *UGT71B6* (**B**), *UGT71B7* (**C**), *UGT71B8* (**D**), *PER30* (**E**), and *RD29A* (**F**) in 10-day-old seedlings treated with 100 μM ABA. Transcript levels were measured by RT-qPCR and normalized to *ACT2* expression levels. Data represent mean values ± SDs (n = 20 seedlings, at least three biological replicates). Significant differences from the no-treatment control (0 h) are indicated by asterisks (*P < 0.05, **P < 0.01, ANOVA followed by Tukey’s honestly significant difference test)

*ELENA19* expression was also induced by ABA treatment. Therefore, we examined whether *ELENA19* affects the expression of neighboring genes via *cis*-regulation by measuring the expression of *ELENA19* and its neighboring genes in WT and *ELENA19* OX lines after 100 μM ABA treatment by RT-qPCR. *ELENA19* was expressed at more than 100-fold higher levels in the OX lines than in Col-0 plants (Fig. 3A). *RD29A* was used as an ABA response marker gene (Fig. 3F and S4). *UGT71B6* expression was significantly down-regulated in the OX lines compared to that in WT plants at early time points after ABA treatment, suggesting that *ELENA19* negatively regulates *UGT71B6* expression in response to ABA (Fig. 3B). *UGT71B7*, *UGT71B8*, and *PER30* expression did not significantly differ between WT and *ELENA19* OX lines at early time points after ABA treatment (Fig. 3C–E). These results suggested that *ELENA19* responds to both PAMP and ABA signaling and specifically down-regulates the expression of its target gene, *UGT71B6*.

### ELENA19 OX plants are sensitive to ABA

**Fig. 4:**
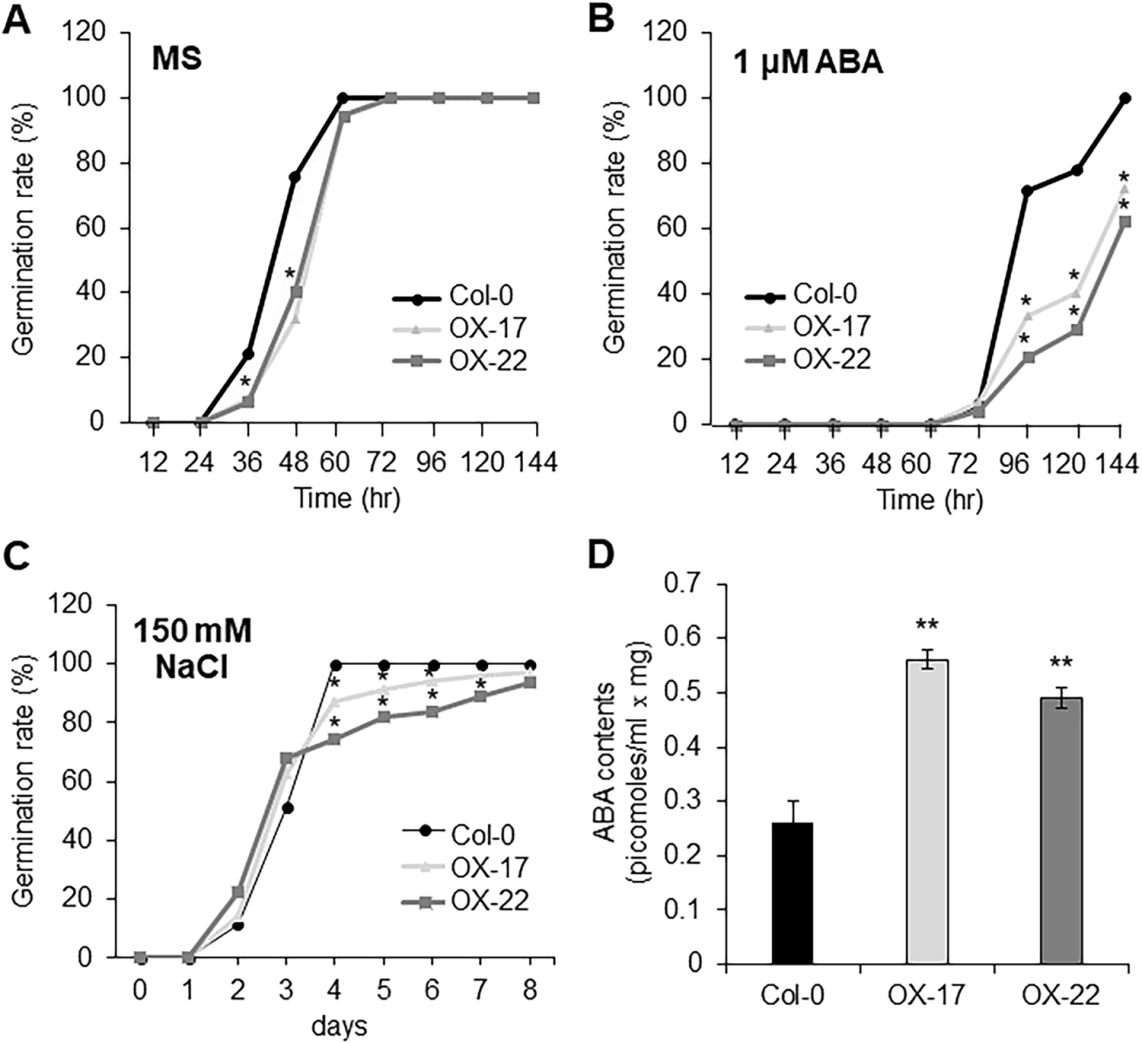
Seed germination rate and ABA content analysis in *ELENA19* OX lines. **A** Germination rates of Col-0 and the OX-17 and OX-22 lines. Seeds were germinated on 1/2 MS plates not supplemented (**A**) or supplemented with 1 μM ABA (**B**) or 150 mM NaCl (**C**). Germination was defined radicle protrusions of at least 1 mm. At least 50 seeds were analyzed in each independent experiment (three replicates). **D** ABA was extracted from 200 mg seedlings (fresh weight). ABA contents were determined by competitive ELISA using an anti-ABA antibody (Phytodetek ABA Test Kit). Significant differences from the WT (Col-0) are indicated by asterisks (*P < 0.05, **P < 0.01, ANOVA followed by Tukey’s honestly significant difference test)

In a previous study, RNAi lines with simultaneous knockdown of *UGT71B6*, *UGT71B7*, and *UGT71B8* were hypersensitive to high-salt stress, but resistant to osmotic stress (Dong et al. 2014). We determined the seed germination rates of the WT and *ELENA19* OX lines without stratification. WT and OX line seeds harvested simultaneously were sown on 1/2 MS medium supplemented or not with 1 µM ABA. After sowing, the germination rate was measured every 12 h at the same time point for 6 days. The seed germination rate of the *ELENA19* OX lines was significantly lower than that of WT plants on standard MS medium (Fig. 4A), and even more so on ABA-containing medium (Fig. 4B). The germination of the *ELENA19* OX lines was also delayed compared to that of WT plants on NaCl-containing medium (Fig. 4C). These results suggested that the overexpression of *ELENA19* increases the sensitivity to ABA. To confirm that the endogenous ABA content was altered by *ELENA19* overexpression, we measured ABA contents in 3-week-old seedlings using a competitive ELISA kit. The results showed that ABA contents were more than two times higher in the *ELENA19* OX lines than in WT plants (Fig. 4D), suggesting that overexpression of *ELENA19* may increase cellular ABA levels. Taken together, these results suggest that *ELENA19* overexpression correlates with increased endogenous ABA levels and enhanced sensitivity to ABA and salt stress, potentially through its regulation of *UGT71B6* expression.

### *ELENA19* is a negative regulator of flg22-dependent callose deposition

**Fig. 5:**
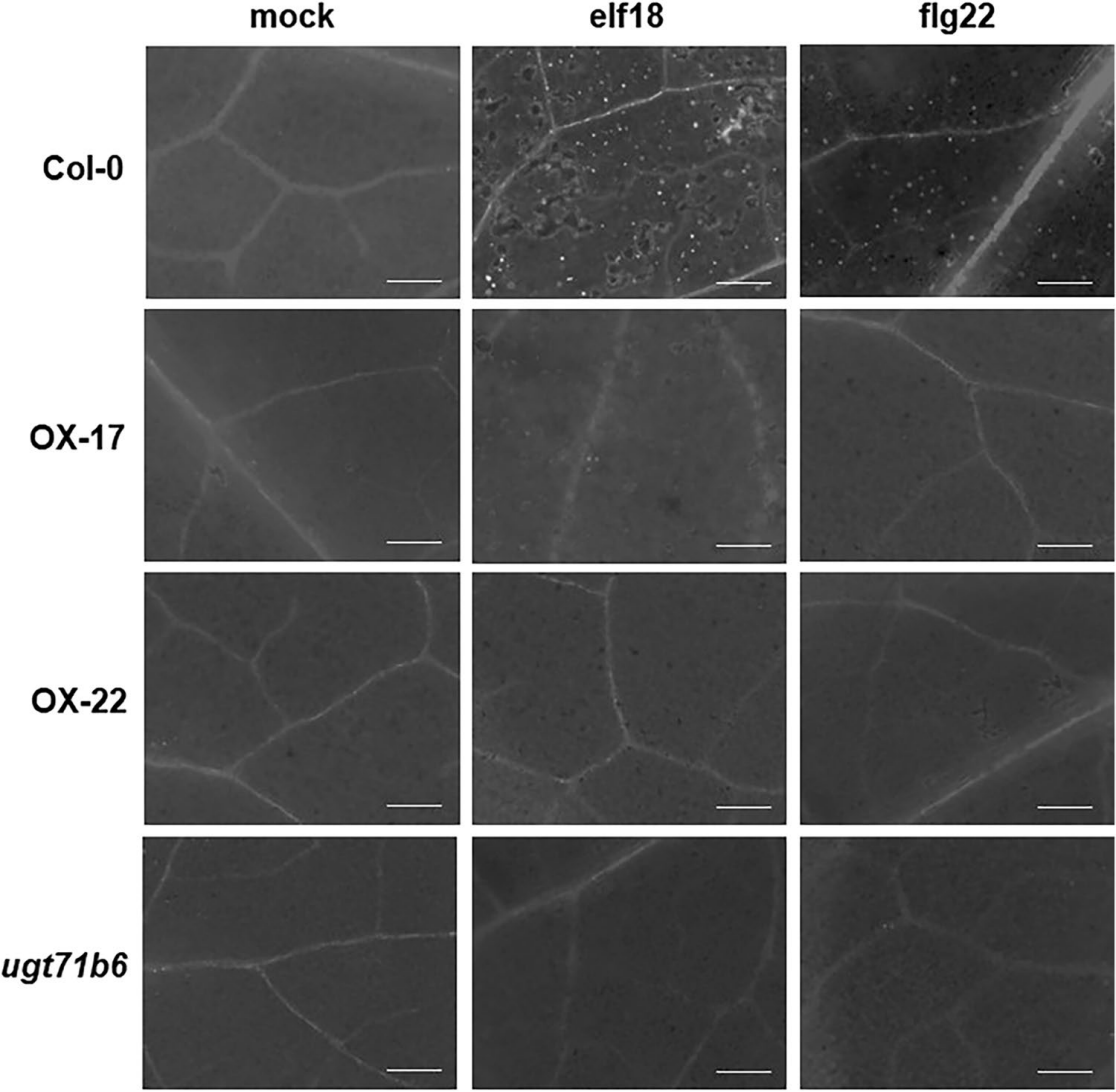
*ELENA19* overexpression suppresses flg22-induced callose deposition. Callose deposition in leaves of Col-0, OX-17, and OX-22 lines and the *ugt71b6* mutant after flg22 treatment. Ten-day-old seedlings were treated with 1 μM flg22 for 24 h, then stained with aniline blue. The white spots indicate callose deposits. The images are representative of 10 seedlings in three independent experiments. Scale bars = 100 μm

Callose deposition has been widely used to quantify plant immune activity. Callose plays an important role in plant defense by slowing down pathogen invasion and spread. We investigated callose deposition in the transgenic and WT plants by aniline blue staining after flg22 and elf18 treatments. As shown in Fig. 5, callose formation was induced in WT plants after flg22 and elf18 treatments. However, in the OX lines, callose deposition was not observed after flg22 and elf18 treatment (Fig. 5). To determine whether the callose-deficient phenotype was caused by *UG71B6* down-regulation, we investigated the *ugt71b6* mutant (SALK_001713) (Figure S5A). Homozygous *ugt71b6* mutation was validated by genotyping (Figure S5B), and RT-PCR confirmed that *ELENA19* and *UGT71B6* were knocked out, whereas *PER30* and *UGT71B7* expression were intact (Figure S5C). The *ugt71b6* mutant also showed the callose-deficient phenotype after flg22 and elf18 treatments. A previous study showed that pretreatment with ABA leads to the abolishment of callose deposition induced by flg22, indicating that ABA negatively regulates flg22-triggered callose formation (Clay et al. 2009). This suggests that increased ABA levels due to *ELENA19* overexpression suppressed flg22-triggered callose formation.

### *ELENA19* down-regulates the expression of ethylene (ET)-dependent flg22-induced genes

**Fig. 6:**
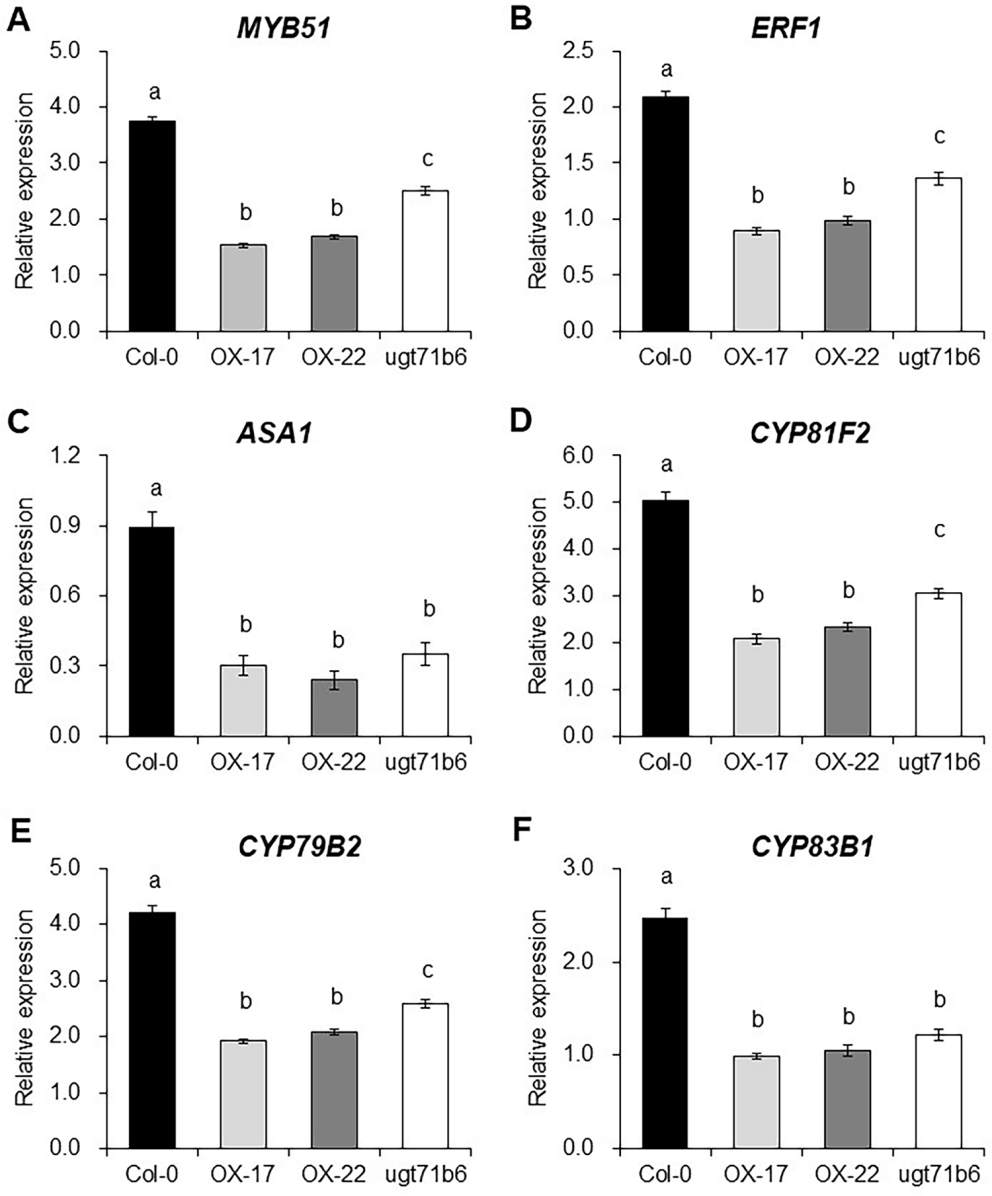
Expression analysis of genes involved in callose deposition in transgenic plants after flg22 treatments. **A**–**F** Relative expression analysis of *MYB51* (**A**), *ERF1* (**B**), *ASA1* (**C**), *CYP81F2* (**D**), *CYP79B2* (**E**), and *CYP83B1* (**F**) in 10-day-old seedlings treated with 1 μM flg22 for 6 h. Transcript levels were measured by RT-qPCR and normalized to *ACT2* expression levels. Data represent mean values ± SDs (n = 10 seedlings, at least three biological replicates). Relative expression levels to the no-treatment control (0 h) were calculated. Different letters indicate significant differences between WT and transgenic plants at *P* < 0.05 (ANOVA followed by Tukey’s honestly significant difference test)

ABA antagonizes ET signaling activated by flg22 and represses the expression of ET-dependent genes. ET signaling is required for the induction of *ERF1*, *MYB51*, *ASA1*, *CYP81F2*, *CYP79B2*, and *CYP83B1*, all of which are essential for flg22-induced callose deposition. *MYB51* is necessary to induce genes involved in indole glucosinolate biosynthesis (*ASA1*, *CYP79B2*, *CYP81F2*, and *CYP83B1*) (Clay et al. 2009). Therefore, we quantified the expression of these genes in *ELENA19* OX and *ugt71b6* plants after flg22 treatment. Expression levels of *ERF1*, *MYB51*, and indole glucosinolate biosynthetic genes (*ASA1*, *CYP81F2*, *CYP79B2*, and *CYP83B1*) were significantly down-regulated in the *ELENA19* OX and *ugt71b6* plants after flg22 treatment compared to the levels in WT plants (Fig. 6). Interestingly, the expression levels of all these genes were also significantly reduced in *ELENA19* OX and *ugt71b6* plants compared to WT plants in the normal physiological state (without flg22 treatment) (Figure S6). This suggests that the increased ABA levels in *ELENA19* OX and *ugt71b6* plants led to the attenuation of gene expression involved in ET-dependent flg22-induced callose deposition. This is consistent with previous findings of antagonistic crosstalk between ABA and ET signaling in flg22-mediated callose deposition (Beaudoin et al. 2000; Clay et al. 2009; Ghassemian et al. 2000).

## Discussion

UGT71B6 and its two homologs mediate the conversion of ABA to its inactive conjugated form, ABA-GE (Dong and Hwang 2014). RNAi lines in which *UGT71B6*, *UGT71B7*, and *UGT71B8* were knocked down simultaneously were hypersensitive to high-salt stress and increased cellular ABA levels (Dong et al. 2014), indicating that the UGT71B family modulates ABA homeostasis. *ELENA19* is a NAT of *UGT71B6* located in the *UGT71B* gene cluster. Therefore, we expected that *ELENA19* could regulate *UGT71B* homologs other than *UGT71B6* given their high nucleotide sequence similarity. However, only *UGT71B6* expression was significantly induced by flg22 and elf18 treatments and was affected by *ELENA19* (Fig. 3), demonstrating that UGT71B6 has a specific role in PTI signaling and that different UGT71B members mediate distinct responses. The transcriptional response of *ELENA19* to flg22, elf18, and ABA was very rapid and reached the maximum level within 30 min after treatments (Figs. 1 and S1). These results suggest that *ELENA19* is involved in flg22-mediated PTI signaling via regulating *UGT71B6* expression in the early stage of the defense response. Moreover, our results demonstrate that *ELENA19* mediates ABA homeostasis and the crosstalk between ABA and PTI through *UGT71B6* regulation. On the other hand, like protein-coding genes, lncRNAs have tissue specificity. We investigated *ELENA19* expression in different developmental stages and tissues of adult (4-week-old) plants. *ELENA19* expression levels were higher in young seedlings and rosette leaves than in the other tissues (Figure S7). Moreover, *ELENA19* was mainly expressed in the leaves and was not detected in stem tissues, suggesting that *ELENA19* plays a particular role in different developmental stages and tissues.

ABA plays a multifaceted role and is involved in a complex network of synergistic and antagonistic crosstalk in plant defense responses. ABA plays different roles in disease resistance depending on the pathogen type, defense response timing, and plant tissue type (Asselbergh et al. 2008; Ton et al. 2009). ABA has been reported to positively regulate both pre- and post-invasion defenses to fungal pathogens. ABA promotes stomatal closure during the early defense stage and induces callose deposition to prevent further invasion after fungal infection (Flors et al. 2008; Kaliff et al. 2007; Ton and Mauch-Mani 2004). Interestingly, the fungal PAMP chitosan enhances ABA synthesis and callose deposition (Iriti and Faoro 2008). ABA also functions as a pre-invasive defense barrier against bacterial pathogens by inducing stomatal closure in the early defense stage. However, ABA plays a negative regulatory role in bacterial pathogen defense in the post-invasion stage. ABA has been reported to suppress bacteria-induced callose deposition and late salicylic acid-induced defense activation (Asselbergh et al. 2008; Ton et al. 2009). Flg22 and elf18 are PAMPs derived from bacterial pathogens, and flg22-induced callose deposition is suppressed by ABA (Clay et al. 2009). Moreover, there is an antagonistic crosstalk between ABA and Flg22-induced ET signaling. ET-dependent Flg22-induced genes, including *ERF1*, *MYB51*, *ASA1*, *CYP81F2*, *CYP79B2*, and *CYP83B1*, were significantly repressed by ABA treatment (Clay et al. 2009). In line herewith, Flg22-triggered callose accumulation was suppressed and the expression of ET-dependent Flg22-induced genes was significantly reduced in *ELENA19* OX plants (Figs. 5 and 6). This is consistent with previously reported antagonistic crosstalk between ABA and ET signaling (Beaudoin et al. 2000; Ghassemian et al. 2000). Therefore, our results support that *ELENA19* mediates the crosstalk between ABA and PTI signaling. Considering that *ELENA19* expression was induced very early, it can be inferred that *ELENA19* is involved in the early stage of defense to prevent pathogen invasion by ABA-induced stomatal closure.

The T-DNA insertion in SALK_001713 disrupts both *UGT71B6* and *ELENA19*, thereby complicating the interpretation of the observed phenotype and making it difficult to attribute the phenotype to a single gene with certainty. Both SALK_001713 and *ELENA19 OX* lines exhibit reduced callose deposition and decreased expression of callose-related genes upon flg22 treatment compared to wild-type plants (Fig. 5 and 6). As *ELENA19* negatively regulates *UGT71B6* expression, its overexpression leads to the down-regulation of *UGT71B6* upon flg22 treatment (Fig. 2B), suggesting that the phenotype observed in *ELENA19 OX* lines is largely mediated through down-regulation of *UGT71B6*. This supports the idea that the loss of *UGT71B6*, rather than disruption of *ELENA19*, is the primary contributor to the phenotype observed in SALK_001713. Accordingly, we have designated SALK_001713 as *ugt71b6*. If the loss of *ELENA19* were primarily responsible, SALK_001713 would be expected to exhibit a flg22-hypersensitive phenotype, characterized by increased callose deposition and upregulation of callose-related genes, opposite to what is observed in *ELENA19 OX* lines. Nonetheless, our data do not exclude the possibility that *ELENA19* influences PAMP-triggered callose deposition through mechanisms that are at least partly independent of *UGT71B6*. This can be directly examined by epistasis analysis (e.g., crossing *ELENA19* OX lines into a *ugt71b6* loss-of-function or knock-down background) and by *UGT71B6* complementation experiments to assess whether the callose phenotype is abolished or restored, respectively.

The regulatory mechanism of *UGT71B6* by *ELENA19* was not clearly identified in this study. *UGT71B6* expression may be down-regulated via RNAi since *ELENA19* is a NAT. However, siRNAs have not been detected in the *ELENA19* locus under normal conditions according to an siRNA-sequencing database (http://epigenomics.mcdb.ucla.edu/smallRNAs/) (Zhang et al. 2007). Moreover, *UGT71B6* expression was moderately increased rather than decreased in *ELENA19* OX plants under normal conditions. If *ELENA19* acts as a precursor for siRNA production, *UGT71B6* expression should be decreased in *ELENA19* OX plants. If this would be the case, the expression levels of *UGT71B7* and *UGT71B8*, the homologous gene of *UGT71B6* with the highest sequence similarity, should also be decreased in an siRNA sequence-dependent manner in *ELENA19* OX plants. However, the expression levels of *UGT71B7* and *UGT71B8* were not significantly decreased in *ELENA19* OX plants after flg22 or ABA treatments.

To reconcile these observations, we note that stimulus-induced *UGT71B6* up-regulation is consistently attenuated in *ELENA19* overexpression (OX) lines following ABA or PAMP treatment, whereas basal *UGT71B6* expression at 0 h is elevated (approximately ~ twofold) relative to wild type. Given these patterns and the current evidentiary limits, we propose two non-mutually exclusive models. First, *ELENA19* may act locally to limit stimulus-evoked transcriptional activation of *UGT71B6*, thereby reducing net transcriptional output specifically during the induction phase. This interpretation is consistent with the reduced *UGT71B6* induction observed after flg22/ABA treatment and with phenotypes associated with elevated free ABA and increased ABA sensitivity in *ELENA19* OX lines. At present, the underlying mechanism remains unresolved. However, potential cis-acting effects of *ELENA19* on *UGT71B6* could include altered local chromatin features and/or constraints on stimulus-dependent transcriptional activation. Second, *ELENA19* may decouple *UGT71B6* from canonical ABA-responsive regulation. Under this model, the elevated basal expression and reduced inducibility of *UGT71B6* observed in *ELENA19* OX lines would reflect a disruption in normal ABA-mediated transcriptional control, rather than direct transcriptional repression by *ELENA19*. This could contribute to ABA accumulation through misregulation of conjugation dynamics. Importantly, active (free) ABA levels reflect the combined flux of biosynthesis, catabolism, conjugation (UGTs), and rapid deconjugation by β-glucosidases (e.g., AtBG1/AtBG2). Under this framework, temporally misregulated *UGT71B6* expression could fail to constrain active ABA pools if conjugation is counterbalanced by stimulus-driven deconjugation and/or increased biosynthetic input, potentially leading to elevated free ABA despite higher basal *UGT71B6* expression.

It is noteworthy that the concurrent induction of *ELENA19*, a negative regulator of *UGT71B6* expression, and *UGT71B6* itself upon PAMP treatment is not an uncommon phenomenon in plants. Similar gene-regulatory dynamics have been well documented across multiple signaling pathways, where negative regulators and their target genes are co-induced in response to specific stimuli. For instance, in the jasmonic acid (JA) signaling pathway, JAZ proteins, transcriptional repressors of JA responses, are induced by JA together with JA-responsive genes that they repress under normal conditions (Chini et al. 2007). Likewise, in the abscisic acid (ABA) signaling pathway, the negative regulators ABI1 and ABI2 are co-induced with ABA-responsive genes following ABA treatment, despite their roles as repressors of ABA signaling (Leung et al. 1997). A comparable expression pattern has also been observed among plant lncRNAs, where negative regulatory lncRNAs are co-induced alongside their target genes. For instance, *cis-NATZmNAC48*, a natural antisense transcript, negatively regulates *ZmNAC48* expression, a transcription factor promoting drought tolerance in maize. Despite its repressive role, *cis-NATZmNAC48* is co-induced with *ZmNAC48* under drought stress conditions (Mao et al. 2021). Collectively, the simultaneous induction of negative regulators and their target genes is a well-established phenomenon in plants. This concurrent induction reflects an evolutionarily conserved regulatory mechanism that enables plants to initiate rapid responses to environmental cues, while also establishing self-regulatory mechanisms to prevent excessive amplification of plant stress signaling. Such expression dynamics are commonly regarded as components of a negative feedback or fine-tuning mechanism, supporting both the robustness and homeostatic balance of plant stress signaling.

In conclusion, *ELENA19* mediates the innate immune response by interacting with ABA homeostasis via regulating *UGT71B6* expression. Our results revealed the complexity of lncRNA roles in transcriptome regulation associated with the plant innate immune response.

## Supplementary Information

Below is the link to the electronic supplementary material.

Supplementary file1 (PDF 269 KB)

Supplementary file2 (XLSX 11 KB)

## Data Availability

All the data in this study was included in the article and supplementary figures.
