# Supplementary material for: ELF18-INDUCED LONG NONCODING RNA 19 attenuates PAMP-induced callose deposition by modulating UDP-glycosyltransferase 71B6-associated ABA levels
Source: Plant Cell Rep. 2026 Jan 19;45(2):35. doi: 10.1007/s00299-026-03720-0 (PMC12816122; doi:10.1007/s00299-026-03720-0)
Supplement: Supplementary file 1 — Supplementary file1 (PDF 269 KB) [file 299_2026_3720_MOESM1_ESM.pdf]

Figure S1

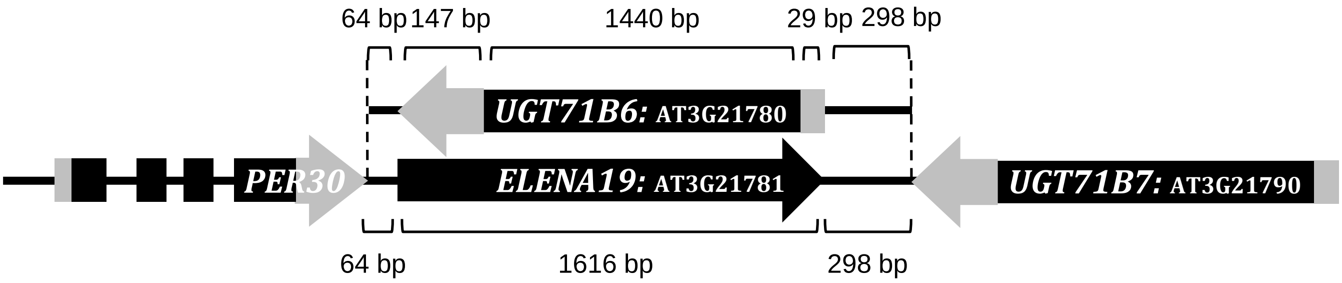

Supplementary Figure 1. Schematic diagram of *ELENA19* and *UGT71B6*. Diagram for location and structure of ELENA19 and its neighboring genes. Gray, untranslated region (UTR); black, exon; numbers, nucleotides.

Figure S2

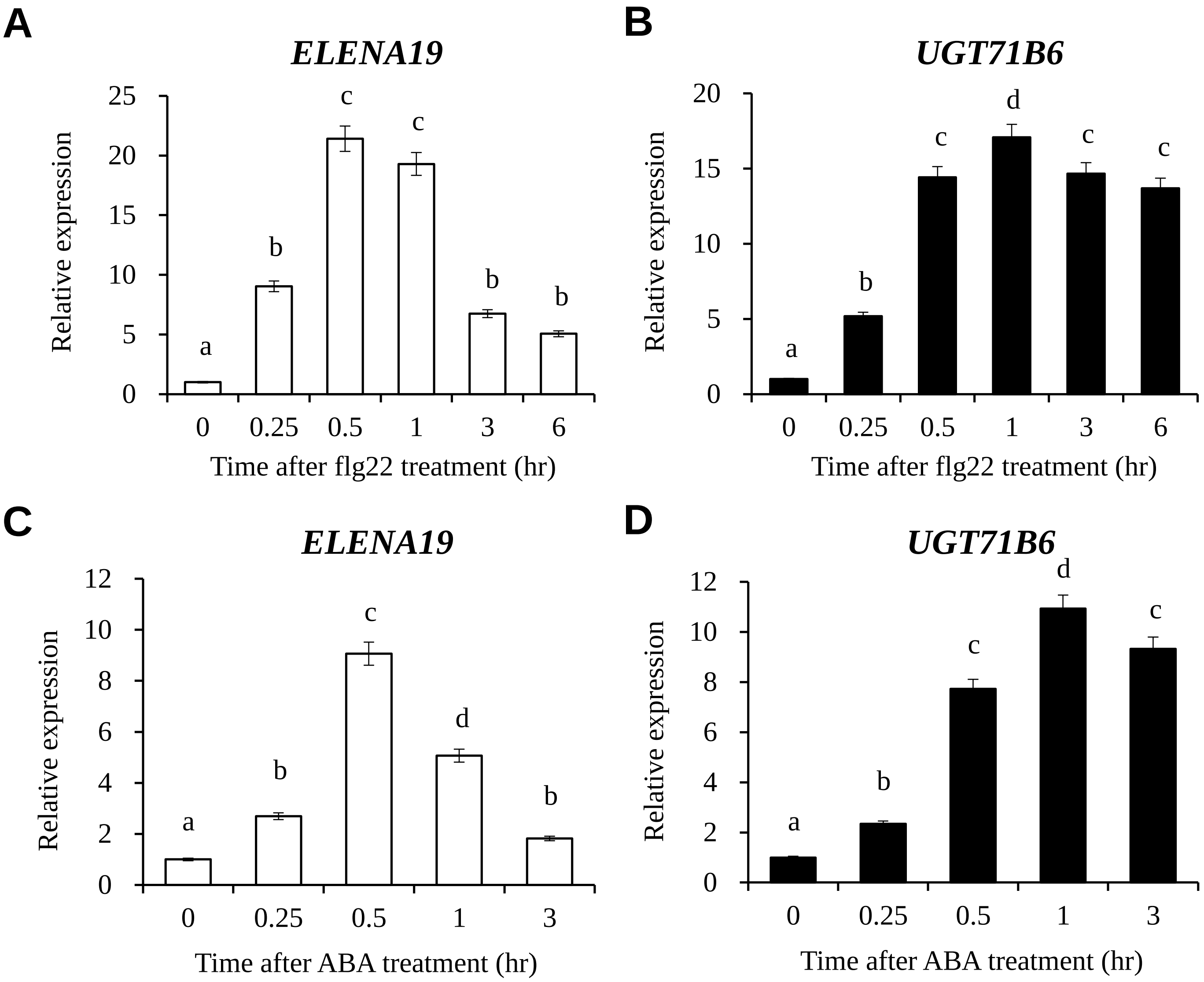

Supplementary Figure 2. *ELENA19* and *UGT71B6* expression are induced early after PAMP or ABA treatment. Relative expression of *ELENA19* (A) and *UGT71B6* (B) after flg22 treatment. Ten-day-old Col-0 (wild type) seedlings were treated with 1μM flg22. Transcript levels were measured by quantitative RT-qPCR normalized with *ACT2* expression levels. Error bars represent average ± SD (n = 20 Arabidopsis seedlings). (B) Relative expression of *ELENA19* (C) and *UGT71B6* (D) after ABA treatment. Ten-day-old Col-0 (wild type) seedlings were treated with 100μM ABA. Transcript levels were measured by RT-qPCR normalized with *ACT2* expression levels. Error bars represent average ± SD (n = 20 Arabidopsis seedlings). Different letters indicate significant differences between non-treated sample and treated sample at $P < 0.05$ (ANOVA followed by Tukey's honestly significant difference)

Figure S3

A

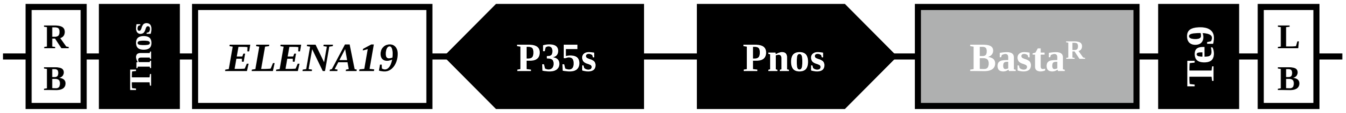

B

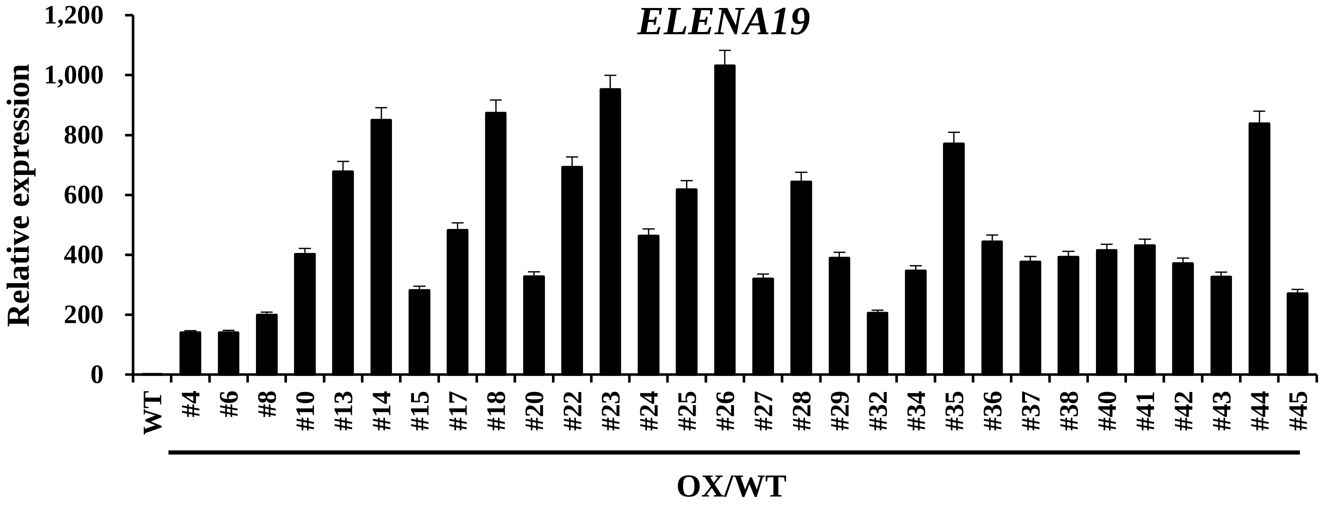

Supplementary Figure 3. *ELENA19* expression levels in the *ELENA19* overexpression lines. (A) Schematic diagram of overexpression vector, pBA-DC construct. LB: left border, Te9: rbcS-E9 terminator, Basta^{R} : bacterial phosphinothricin acyl-transferase gene (a selectable marker), Pnos: the nopaline synthase promoter, P35S: Cauliflower mosaic virus 35S promoter, *ELENA19*: annotated transcript of *ELENA19*, Tnos: the nopaline synthase polyadenylation region (terminator), RB: right border (B) Relative expression of *ELENA19* in T1 overexpression plants. Leaf discs were used for RNA extraction. Transcript levels were measured by RT-qPCR and normalized with *ACT2* expression.

Figure S4

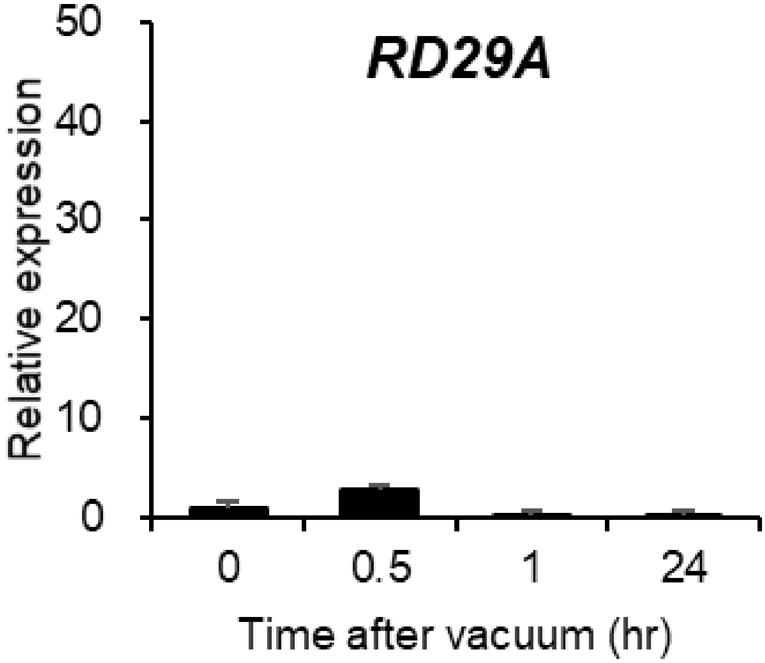

Supplementary Figure 4. Expression analysis of *RD29A* in WT plants after vacuum infiltration. Time-course expression analysis of *RD29A* in 10-day-old seedlings treated with vacuum for 5 min. Transcript levels were measured by RT-qPCR and normalized to *ACT2* expression levels. Data represent mean values ± SDs (n = 20 seedlings, at least three biological replicates).

Figure S5

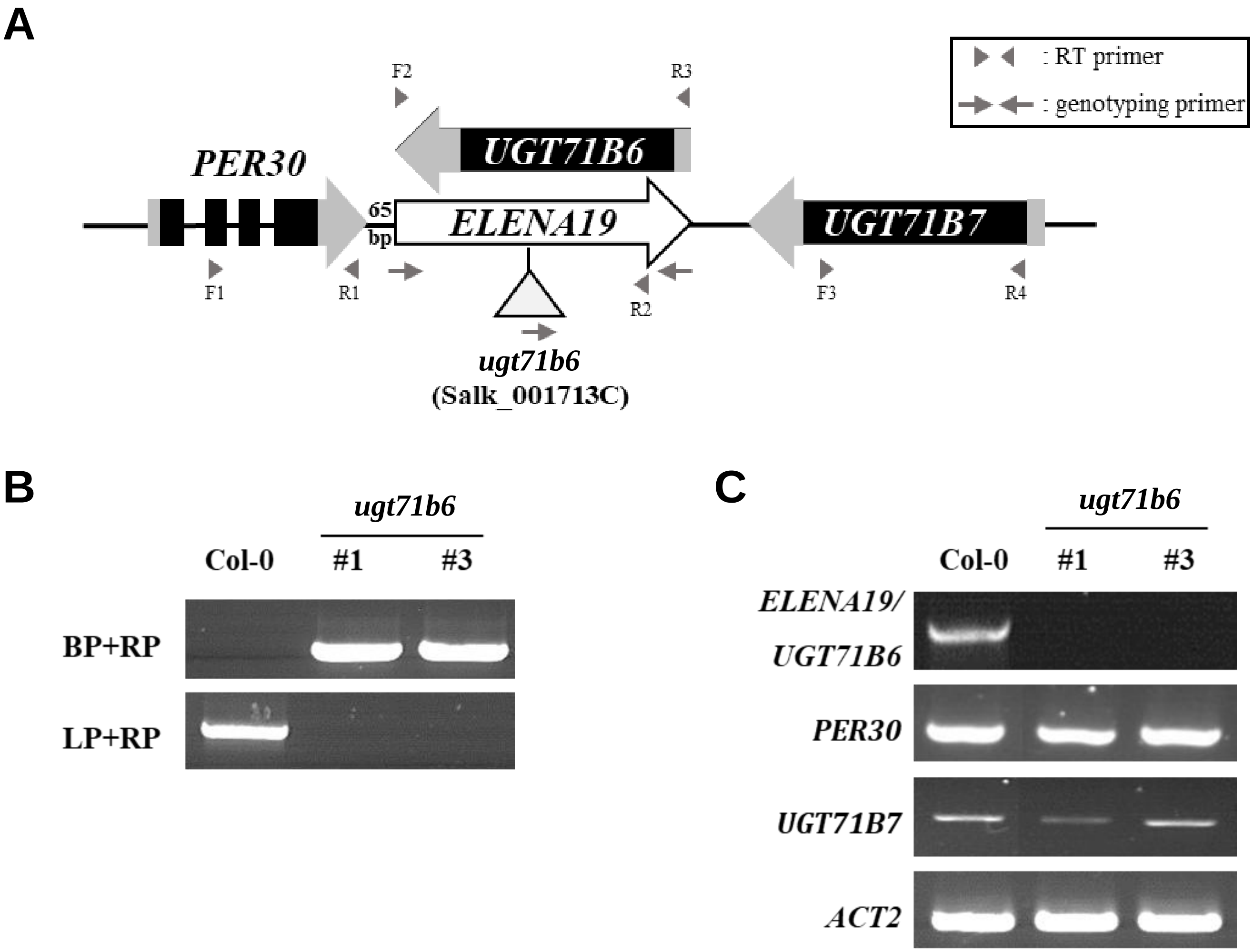

Supplementary Figure 5. Validation of the *ugt71b6* knockout mutant. (A) Schematic representation of T-DNA insertion site. A gray triangle indicates T-DNA insertion of *ugt71b6* (SALK_001713C). Gray and black boxes show untranslated regions and exons, respectively. Solid lines represent introns or intergenic regions. Gray arrowheads indicate primers for RT-PCR. Gray arrows indicate primers for genotyping. (B) Identification of *ugt71b6* (SALK_001713C) by genotyping PCR. Primers used for genotyping PCR were recommended from SIGnAL, iSect primer design tool. (C) Identification of *ugt71b6* (SALK_001713C) by RT-PCR. RT-PCR primers were designed to be specific to *ELENA19*, *PER30*, and *UGT71B7* sequence based on the microarray result and NCBI blast. cDNA was synthesized using oligo(dT)$_{20}$. *ACT2* was used as an internal control.

Figure S6

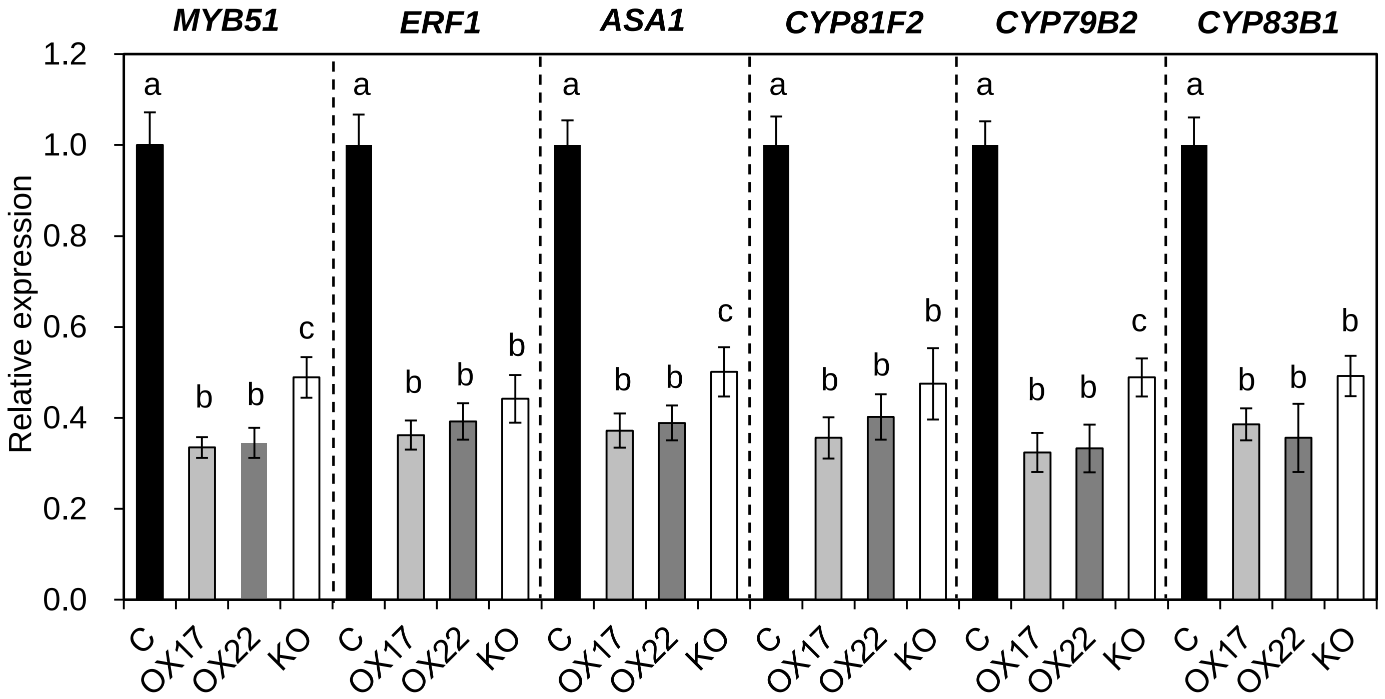

Supplementary Figure 6. Expression analysis of genes involved in callose deposition in transgenic plants under normal physiological conditions. Relative expression analysis of *MYB51*, *ERF1*, *ASA1*, *CYP81F2*, *CYP79B2*, and *CYP83B1* without treatment. Ten-day-old seedlings were used for analysis. Black, light gray, dark gray, and white bar represent Col-0 (C), *35S:ELENA19* #17 (OX17), *35S:ELENA19* #22 (OX22), and *ugt71b6* (KO), respectively. Transcript levels were measured by RT-qPCR and normalized with *ACT2* expression levels. Data represent mean values ± SD (n = 10 seedlings, biological repeat > 3). The relative expression level was calculated from no-treatment control (0 hr). Different letters indicate significant differences between WT and transgenic plants at $P < 0.05$ (ANOVA followed by Tukey's honestly significant difference).

Figure S7

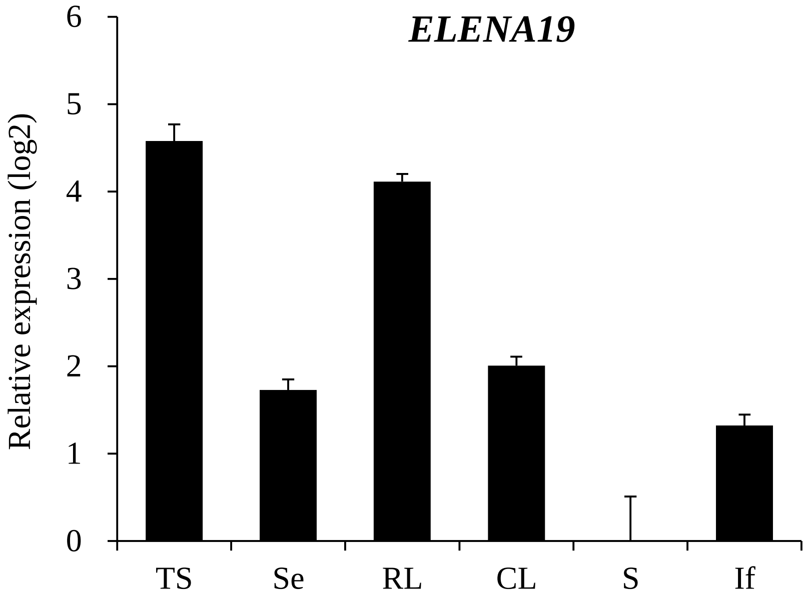

Supplementary Figure 7. *ELENA19* expression levels in different growth stages and tissues. Total RNA was extracted from various growth stages and organs: three-day-old seedling (TS), ten-day-old seedling (Se), rosette leaves (RL), cauline leaves (CL), stem (ST) and inflorescence (If). Three-day-old and ten-day-old seedlings were grown in 1/2 MS medium and the rest of the organs were extracted at four-week-old mature plants grown on soil. Transcript levels were measured by RT-qPCR and normalized with *ACT2* expression. Each bar represents the log2 value of fold change by the lowest expressed organ detected. Error bars represent average ±SD (n = 10, three independent experiments)
